# Serological and molecular detection of *Toxoplasma gondii* in naturally infected red foxes (*Vulpes vulpes*) from Victoria, Australia

**DOI:** 10.1007/s00436-025-08606-w

**Published:** 2025-12-16

**Authors:** Leonardo Brustenga, K. L. D. Tharaka D. Liyanage, Panayiotis Loukopoulos, Megan Fisher, Jessica Haining, Charles G. Gauci, Livia Lucentini, Alessandro D. Uboldi, Christopher J. Tonkin, Giulia Morganti, Giulia Rigamonti, Fabrizio Passamonti, Fabrizia Veronesi, Abdul Jabbar, Jasmin Hufschmid

**Affiliations:** 1 Department of Veterinary Medicine, University of Perugia, Perugia, Italy; 2 Department of Veterinary Biosciences, Melbourne Veterinary School, Faculty of Science, The University of Melbourne, Werribee, VIC Australia; 3 Department of Chemistry, Biology and Biotechnology, University of Perugia, Perugia, Italy; 4 Department of Medical Biology, The University of Melbourne, Parkville, VIC Australia; 5 The Walter and Eliza Hall Institute of Medical Research, Parkville, VIC Australia

**Keywords:** *Toxoplasma gondii*, Red fox, *Vulpes vulpes*, Australia, Victoria

## Abstract

*Toxoplasma gondii* is a zoonotic parasite with a global distribution that can infect a wide range of warm-blooded hosts. This study investigated, for the first time, the seroprevalence and genetic variability of *T. gondii* in red foxes (*Vulpes vulpes*) from Victoria, Australia. Animals from both regional Victoria and the metropolitan Melbourne area were sourced from trappers and shooters involved in pest control. Sera were screened for anti-*T. gondii* IgG antibodies using a modified agglutination test, and tissue samples were tested using a qPCR assay targeting the 529 bp repeated element. qPCR positive samples were genotyped using a five-marker (L358, 5’SAG2, 3’SAG2, c22-8, GRA6) polymerase chain reaction-restriction fragment length polymorphism protocol. Anti-*T. gondii* antibodies were detected in 38.9% (30/77) of foxes, and parasite DNA was identified in 23.4% (18/77) of animals. Genotyping revealed a predominance of *T. gondii* clonal Type II whereas two isolates showed new alleles attributable to Type II-like genotypes. These findings suggest that Australian red foxes are frequently exposed to *T. gondii* and may play an important role as epidemiological sentinels to assess the circulation of *T. gondii* in the Australian environment, with implications for both wildlife conservation and public health.

**Supplementary Information:**

The online version contains supplementary material available at 10.1007/s00436-025-08606-w.

## Introduction

The red fox (*Vulpes vulpes* Linnaeus, 1758) is a resilient and highly adaptable canid with the widest geographical distribution of any wild carnivore (Maconald and Sillero-Zubiri 2004). According to IUCN’s Invasive Species Specialist Group, the species is listed among the 100 most dangerous invasive alien species (Lowe et al. 2000). Native to the northern hemisphere, red foxes were introduced to Australia from Great Britain throughout the 19th century (Fairfax 2019), but release events in southern Victoria in the 1870 s are thought to be responsible for the source populations that allowed for the colonisation of the whole continent (Rolls 1969). Invasive foxes have contributed to the extinction and decline of many native species (Woinarski et al. 2019, 2021; Stobo-Wilson et al. 2021).

Aside from their direct impact on Australian native fauna, foxes can carry an extensive range of viral, bacterial and parasitic pathogens that can pose threats to animals and humans (Saunders et al. 2010). The impact of the spread of parasites by foxes has been highlighted for several pathogen groups, the majority of which are zoonotic. For example, *Spirometra erinaceieuropaei*, a tapeworm, can infect a wide range of vertebrates, including humans, and cause sparganosis, a debilitating disease associated with the migration of the larval stage inside the host tissues (Newsome and Coman 1989). Similarly, *Echinococcus granulosus*, the causative agent of cystic echinococcosis, poses a major risk to humans and livestock due to the development of hydatid cysts in the brain and visceral organs (Jenkins and Morris 2003). Foxes may also become infected with the apicomplexan protozoan *T. gondii*; however, while the parasite may impact on the health and behaviour of its hosts, including many domestic and wild species, as well as humans, foxes, being higher order predators, are generally a dead-end host.

Genetic analyses based on restriction fragment length polymorphism (RFLP) of *T. gondii* isolates from across the world revealed the presence of three main clonal lineages, referred as type I, type II and type III (Ajzenberg et al. 2004; Shwab et al. 2014; Dubey 2021). Advancements in the number of isolates processed and the number of loci analysed allowed for the recognition of 343 different genotypes, now referred as ToxoDB genotypes, along with the three archetypal genotypes that are grouped in six different clades (Su and Dubey 2020; Dubey 2021; Valenzuela-Moreno et al. 2025). Type II and III genotypes are dominant in Africa and the Northern Hemisphere, while more genetically diverse isolates, referred as atypical strains have been detected alongside the clonal lineages, particularly in South America where genomic recombination by sexual reproduction of the parasite is thought to be much higher (Shwab et al. 2014; Galal et al. 2019). Phylogenetic analyses of *T. gondii* from wildlife in Australia were primarily carried out on macropods, with non-archetypal type II-like and atypical strains frequently reported in several species (Liyanage et al. 2025).

While foxes, like other canids (Morganti et al. 2024), are not thought to be a significant contributor to the spread of *T. gondii*, because they do not shed infectious oocysts and do not have many predators, their omnivorous diet makes them excellent epidemiological sentinels to assess the presence of the parasite in the environment (Bouchard et al. 2022). The use of sentinel species provides us with information on the extent of the circulation of *T. gondii* in the environment, which is of significant importance. However, there are no published estimates of the prevalence of *T. gondii* in Australian red foxes, an introduced and invasive species.

The aim of this study was to conduct a serological and molecular survey of the prevalence of *T. gondii* in red foxes in Victoria. Opportunistically obtained samples were tested using an in-house modified agglutination test (MAT) and a qPCR assay, followed by the genotyping of the four *T. gondi* genes (SAG2, L358, c22-8, GRA6) using an in-silico polymerase chain reaction-restriction fragment length polymorphism (PCR-RFLP) approach.

## Materials and Methods

### Study area and carcass collection

Red fox carcasses were opportunistically collected from hunters and pest control professionals under the framework of Victorian pest animal control containment from November 2024 to January 2025. All trapping and shooting procedures were performed by trained and authorised personnel working independently to this study. Carcasses were collected from urban, suburban and rural settings across the state. Postcodes for each fox were recorded to use as the location of provenance and later all animals were categorised into two groups: “Metropolitan”, according to the borders of metropolitan Melbourne regions identified by Victoria’s Department of Environment, Land, Water and Planning (https://www.planning.vic.gov.au/data/assets/pdf_file/0026/628361/plan-melbourne-map-metro-melbourne-regions.pdf accessed on 09/11/2025); and “Regional”, which included all animals sampled outside the metropolitan area. After being killed, foxes were put in plastic bags and then brought to the University of Melbourne, Werribee campus, where they were stored at 5° C until further processing. All carcasses were processed within 24 h of death. Consultation with Australian authorities confirmed that animal ethics approval and wildlife research permits were not required as (i) foxes were deceased from reasons outside the scope of this study and (ii) since red foxes are a declared pest species in Australia no permit is needed to obtain or retain samples from these animals or the animals themselves.

### Autopsy and sample collection

A complete autopsy was carried out for each of the carcasses. Along with an identification number, four parameters were noted for each animal: location, sex, body mass and age class. The body mass of each fox was measured to the nearest 100 g using scales (Salter Brecknell Model 180). Age was categorised into juvenile and adult, based on incisor teeth eruption and wear (Harris 1978), and body mass. Individuals weighing less than 3.5 kg and showing deciduous teeth were classified as juveniles while those under 3.5 kg with permanent or worn teeth, as well as all individuals over 3.5 kg, were classified as adults (Cavallini 1996). This combined approach, using both dentition wear and body mass for age class estimation, minimised potential bias arising from the classification system being affected by animals with damaged teeth and also accounted for variation in the nutritional status of the animals by not relying solely on body mass.

Blood samples were collected as whole blood from the heart or blood mixed with thoracic fluid in 1.5 mL Eppendorf tubes. All blood samples were centrifuged at 14,000 rpm for 30 min to pellet as much cellular debris as possible, and the supernatant was transferred to a new microcentrifuge tube. Ten grams of pooled tissues (brain stem, tongue, heart, diaphragm, liver and skeletal muscle) were finely minced with sterile scalpel blades and collected in 15 mL Falcon tubes. Only a fragment of the brainstem was collected as the whole brain was harvested for another research project, and skeletal muscle was collected from the proximal end of the biceps femoris. Blood and tissues were stored at −20 °C until further processing. To avoid cross-contamination of samples, all instruments, as well as the dissection tables, were thoroughly cleaned before and after each dissection with a commercial chlorinated sanitiser, FoamChlor (Envirochem), containing < 10% potassium hydroxide and < 10% sodium hypochlorite. The sanitised surfaces and instruments were thoroughly rinsed with water to avoid PCR inhibition caused by lingering leftover sanitiser.

### Serological testing

An in-house MAT previously developed and validated (Liyanage et al. 2024) was used to test fox blood samples for the presence of anti-*T. gondii* IgG. The MAT reactions were performed in round-bottomed 96-wells microtiter plates (Greiner Bio-One GmbH, Kremsmünster, Austria), 1:25 dilutions of the samples were obtained by mixing 3 µL of blood samples with 72 µL of phosphate buffered saline (PBS) and were used as lower cut-off before performing four 2-fold serial dilutions to titre the samples up to 1:200. Positive and negative controls were added to each plate and were titrated up to 1:3200. In-vitro cultured *T. gondii* tachyzoites were used as antigen source preparing an antigen mixture composed of 2.55 mL of alkaline storage buffer (pH = 8.95), 35 µL of 2-mercaptoethanol (Sigma-Aldrich, Missouri, United States), and 150 µL of *T. gondii* tachyzoite suspension. The antigen mixture was briefly vortex mixed and 25 µL were aliquoted in each plate well and mixed to the samples by repeated pipetting. Plates were then sealed and incubated at 37 °C for 12 h. After incubation, wells with a clear and defined pellet dot on the bottom of the well were considered as negative, whereas wells with a diffuse mat covering more than half of the well bottom were considered as positive. Serological positivity was defined using a cut-off MAT titre $\:\ge\:$1:25.

### Molecular testing

Total genomic DNA was extracted from the tissues using Qiagen’s DNeasy blood and tissue kit (Qiagen, Hilden, Germany) following a modified protocol (Liyanage et al. 2025). Before DNA extraction, tissues were thoroughly homogenised to disrupt the parasitic tissue cysts. The homogenised tissues were then vortex-mixed and 300 µL of homogenate were transferred to a 1.5 mL Eppendorf tube along with 300 µL of Buffer AL. Samples were vortex-mixed and incubated at 70 °C for 10 min. Samples were centrifuged at 14,000 rpm for 1 min to pellet all the cellular debris. The supernatant (380 µL) was recovered and transferred to another 1.5 mL tube containing 200 µL of absolute ethanol. Samples were vortex-mixed and the whole sample was loaded in the spin column apparatus. From here on, extraction was carried out according to the manufacturer’s protocol, eluting the DNA in a final volume of 200 µL.

The extracted DNA was used as a template for a qPCR to detect *T. gondii*’s 529 bp (bp) highly repetitive element using a TaqMan probe-based assay (Homan et al. 2000) carried out in a Qiagen Rotor-Gene Q thermal cycler (Qiagen, Hilden, Germany). To detect possible PCR inhibition, an internal control using Equine Herpes Virus (EHV) DNA was amplified using a TaqMan probe-based assay (Liyanage et al. 2025). The sequences of the primers and probes are provided in Table S1. All samples were run in duplicates, and positive and non-template controls were added to each qPCR run. Samples were deemed as positives only if the Ct values, averaged between the two duplicates, were less than or equal to 33 cycles (Liyanage et al. 2025).

### *Toxoplasma gondii* genotyping

An in-silico PCR-RFLP protocol (Su et al. 2010) was used to genotype qPCR-positive samples targeting five selected markers: 3’SAG2; 5’SAG2; GRA6; c22-8; L358 (Liyanage et al. 2025). Each isolate was firstly amplified in a multiplex PCR (mPCR) using a mix of external primers for each gene, a 1:1 dilution was made and used as template for five different nested PCRs (nPCR) using internal primers. The sequences of external and internal primers are given in Table S2. Amplification products were checked for band presence and expected product size on a 1.5% agarose gel stained with GelRed Nucleic Acid Gel Stain (Biotium Australia) in 1.5x TBE buffer using a 100 bp DNA ladder (Promega). Samples that produced bands of expected size for at least three of the five genes were outsourced for purification and bidirectional sequencing to Macrogen Inc. (South Korea). All sequencing data were handled with MEGA v.11 (Tamura et al. 2021) in which a sequence alignment for each gene was produced. Electropherograms were visually inspected to rule out double-peaks and to correct wrong base-calls that could prevent the correct functioning of the in silico RFLP protocol. Nucleotide BLAST (nBLAST) was used to determine the taxonomic identity of each sequence, while virtual enzymatic restrictions were performed by uploading the sequences to the online software Molbiotools Restriction Analyzer (https://molbiotools.com/restrictionanalyzer.php accessed on 24/02/25) and selecting the proper restriction enzymes for each gene (Su et al. 2010): *Hha*I for 3’SAG2; *Mbo*I for 5’SAG2; *Mse*I for GRA6; *BsmA*I and *Mbo*II for c22-8; and *Hae*III and *Nla*III for L358. Reference sequences for genotypes I (RH), II (ME49) and III (CTG) were derived from Liyanage et al. (2025) and used to compare the RFLP patterns.

### Statistical analysis

A multivariable logistic regression model guided by a Directed Acyclic Graph (DAG) was carried out to assess both the effect of the different variables on *T. gondii* seropositivity and possible inter-variable effects. DAGitty (https://www.dagitty.net/ accessed on 17/02/25) was used to generate the DAG, detailed justifications for each causal link are provided in the supplementary materials (SM1), multivariable logistic regression analyses were carried out using Jamovi (https://www.jamovi.org accessed on 17/02/25) statistical software. The interpretation of the observed associations was based on consideration of both 95% confidence intervals (95% CI) and biological relevance.

## Results

### Sample characteristics 

A total of 77 foxes were collected throughout the three months of sampling: 25 (32.5%) from regional Victoria (Fig. S1a, c) and 52 (67.5%) from metropolitan Melbourne (Fig. S1b, d). Male foxes (44/77, 57.1%) were slightly more abundant in the sample compared to females (33/77, 42.9%); 46 out of 77 foxes (59.7%) were categorised as adults (18 females and 28 males), whereas 31 (40.3%) were categorised as juveniles (15 females and 16 males).

### Seroprevalence of *T. gondii*

Overall, 30 out of 77 foxes (38.9%, 95% CI 28.8–50.1) tested positive for anti-*Toxoplasma gondii* antibodies by MAT. Of the 30 positive animals, 11 (36.7%) were females and 19 (63.3%) were males, whereas 9 (30%) were juveniles and 21 (70%) were adults.

### Risk factor analysis

**Fig. 1:**
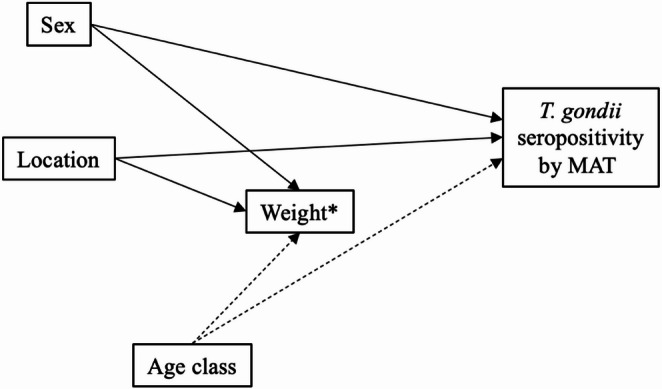
Directed Acyclic Graph (DAG) accounting for each putative causal path linking each of the considered variables to the modified agglutination test (MAT) outcome for the screening of *Toxoplasma gondii* seroprevalence in Australian red foxes (*Vulpes vulpes*). A detailed justification for each of the causal paths is provided in Supplementary Material 1. *Weight used as a proxy to give an estimate of age class Seroprevalence based on age class, sex and location, odds ratios (O.R.) with 95% confidence intervals and relative p-values; and the required adjustment for each variable are shown in Table 1

The DAG-guided logistic regression analysis showed the need for adjustments for just one (age class) of the four variables analysed (Fig. 1).

**Table 1:** Directed acyclic graph (DAG)-guided logistic regression analysis outputs for four variables associated with seropositivity to *Toxoplasma gondii*. Adjusted crude odds ratios (OR) with relative 95% confidence intervals (C.I.) and crude p-value were based on the adjustments advised by the DAG shown in Fig. 1, and are relative to the following reference categories: juvenile, female and rural

Variable	Categories	Seroprevalence (*n*/*N*)	OR (95% C.I.)	*p*-value	Adjusted OR (95% C.I.)	Adjusted *p*-value	Adjustment set
Body mass	N/A	N/A	1.51 (1.11–2.06)	**0.010**	1.62 (1.12–2.35)	**0.010**	Sex, Location
Age class	Adult	45.65% (21/46) 29.03% (9/31)	2.05 (0.78–5.41)	0.145	2.06 (0.77–5.52)	0.150	Sex, Location
Sex	Male	43.18% (19/44) 33.33% (11/33)	1.52 (0.60–3.90)	0.382	1.52 (0.60–3.90)	0.382	No adjustment needed
Location	Metro	40.38% (21/52) 36.00% (9/25)	1.20 (0.45–3.23)	0.712	1.20 (0.45–3.23)	0.712	No adjustment needed

Significant p-values are highlighted in bold

N/A: Not Applicable

n/N: Number of *T. gondii* positive foxes/Number of total foxes in the category

According to the DAG-guided logistic regression analysis, each kilogram increase in body weight was associated with a 1.62 increase in the odds of a fox being seropositive to *T. gondii* (CIs: 1.12–2.35). While the result was not statistically significant, male foxes tended towards a higher seroprevalence compared to females. The odds of seropositivity were comparable between foxes from metropolitan Melbourne and regional Victoria (OR = 1.20, 95% CIs: 0.45–3.23)

### Molecular detection of *Toxoplasma gondii*

*Toxoplasma gondii* DNA was detected by qPCR in 18 (out of 77) foxes (23.4%, 95% CIs: 15.3–34), with an average Ct value of 28.39 (24.54–32.89). All samples that tested positive on qPCR also tested positive on MAT (18/18; 100%); of the qPCR samples that tested negative, 12 had been positive on MAT (12/77; 15.6%), whereas 47 tested negative on both qPCR and MAT. An overview of all the samples that tested positive on MAT and their relative qPCR Ct values is presented in Table S3. Out of the 18 samples, only 13 produced amplicons of expected size and of acceptable quality for sequencing in at least three of the five selected genes. Upon sequence analysis, just 7 out of the 13 sequenced samples produced sequences that were qualitatively satisfactory and identified as *T. gondii* for at least three genes.

### Genotyping and sequence analysis

Genotype assignment was based on the availability of a minimum of three genes to enhance the reliability of the results. The results of genotyping for each of the five genes are reported in Table 2

**Table 2:** Genotyping results for the five genes across all samples tested in the PCR-RFLP protocol to genotype *T. gondii* in tissue samples from red foxes (*Vulpes vulpes*) in Victoria, Australia. The samples that produced at least three reliable sequences are highlighted in bold

Sample ID	3’SAG2	5’SAG2*	c22-8	GRA6	L358
**Vv01**	-	Type I/II	-	Type II	Type II
**Vv03**	Type II	Type I/II	Type II	Type II	Type II
Vv13	-	Type I/II	-	-	Type II
Vv14	Type II	-	-	-	-
Vv17	Type II	-	Type II	-	-
Vv19	-	Type I/II	-	-	-
**Vv24**	Type II	Type I/II	Type II	Type II	Type II
**Vv25**	Type II	Type I/II	Type II	Type II	Type II
**Vv26**	Type II	Type I/II	Type II-like^†^	-	Type II
**Vv38**	Type II	Type I/II	Type II	Type II	Type II
**Vv39**	Type II	Type I/II	Type II	-	Type II
Vv54	-	-	-	Type II	Type II-like^†^
Vv65	Type II	Type I/II	-	-	-

*: 5’SAG2 gene is not able to differentiate between Type I and Type II; newly detected polymorphisms that did not impair the RFLP protocol was detected in all the sequences of the 5’SAG2 gene

^†^: sequences that included newly detected polymorphisms that did not impair the RFLP protocol

All sequences were deposited in GenBank under the accession numbers PX493417-PX493423. A single polymorphism was detected in the sequence of the c22-8 gene for sample Vv26, with a G to A substitution in position 454, resulting in an amino acid substitution from glutamic acid (GAA) to lysine (AAA). In addition, two polymorphisms were detected in the sequence of the L358 gene for sample Vv54; these included a G to A substitution in position 258 and a G to T substitution in position 346. The former represents a silent mutation as both the original and substituted codons code for the amino acid proline (CCG, CCA); the latter represents the substitution of a stop codon (TAG) for glutamic acid (GAG). Along with the aforementioned mutation sites, all sequences for the 5’SAG2 gene showed a double peak (T + A) in position 12 in both the forward and reverse sequences, typical of Type I and II (T) and Type III (A). None of the mutations detected arose in the cleaving sites of restriction enzymes; therefore, the RFLP protocol was not impaired.

## Discussion

This study provides the first evidence of *T. gondii* prevalence and genetic diversity in naturally infected red foxes in Australia. We report a high infection rate, dominated by genotype II and II-like strains.

The detected seroprevalence of *T. gondii* in Australian red foxes (38.9%) was consistent with the global seroprevalence of 39.6% (*n* = 7995), reported in a meta-analysis that examined 40 studies from around the world (Wei et al. 2021). Interestingly, the studies surveyed in the meta-analysis found that sex is not a significant risk factor for *T. gondii* infection in red foxes (Wei et al. 2021). Although the present study also did not find a significant difference in seroprevalence based on sex, males tended towards a higher seroprevalence (63.3%) than females (36.7%).

The prevalence of infection with *T. gondii* in this sample of foxes suggests a comparable level of exposure compared to the definitive host, even though foxes have a wider dietary breadth actively eating plant material and carcasses thus being theoretically more exposed to the parasite. Domestic cats are the only definitive host for *T. gondii* in Australia and are therefore the only source of oocysts. Two continent-wide studies on the prevalence of *T. gondii* in domestic (Brennan et al. 2020) and feral/stray (Liyanage et al. 2024) cats from Australia were recently carried out. Both studies found seroprevalence of *T. gondii* to be relatively high, with 39% (162/417) and 40.4% (223/552), respectively, similar to the results obtained in this study. Key risk factors for infection in domestic cats were outdoor access and raw meat diets (Brennan et al. 2020), whereas the infection status of feral and stray cats was influenced by body weight, geographic location, season and lifestyle (feral vs. stray) (Liyanage et al. 2024). Given that foxes and feral/stray cats are present in the same environments, and that red foxes have at least as broad a dietary spectrum as cats, it is reasonable to believe that the two species share the same risk factors for *T. gondii* infection. The lack of statistical significance for risk factors such as age class or geographical location in the present study could thus be attributed to the relatively small sample and the narrow sampling timeframe. Red foxes are also much more reliant on scavenging of both large animal carcasses and human garbage compared to cats, which are obligate predators of small animals. This generalist foraging could be linked to an increased probability of infection with *T. gondii* in red foxes.

Unsurprisingly, the prevalence of infection based on molecular testing of tissues for the presence of parasite DNA was lower than the seroprevalence estimate. This finding was expected and can be attributed to the stochastic possibility of not including *T. gondii* in selected tissue samples, as the parasite’s cysts are not uniformly distributed in host tissues (Dubey 2021). Furthermore, just a small fragment of the brainstem could be sampled, as the whole brain was fixed in formalin for a parallel research project. Nonetheless, all samples in which *T. gondii* DNA was detected were also positive on serology. Serological methods are more sensitive tools to diagnose infection with *T. gondii* (Kornacka et al. 2016; Adriaanse et al. 2020). They are also more suitable for mass screenings and for assessing exposure to the parasite at the population level. While molecular analyses provide valuable information about the parasite, the practical and methodological constraints outlined above make them less practical diagnostic tools for mass screenings.

Overall, only 7 out of the 18 (38.9%) samples that tested positive to qPCR produced sufficient quality amplicons to be used in the RFLP genotyping protocol. This disparity of results was yet again to be expected, especially for samples that tested positive with high Ct values near the threshold value. It has been demonstrated that the qPCR assay targeting the 529 bp repetitive element is considered to be 10 to 100 times more sensitive than conventional PCR assays (Liyanage et al. 2025; Su et al. 2010). All samples sequenced belonged to the genotype II, the most common genotype detected in red foxes worldwide (Wei et al. 2021) as well as the most common genotype found in Australian marsupials (Dubey 2021) and domestic cats (Brennan et al. 2016). Three sequences presented unique mutations. The most interesting finding is the concurrent presence of a thymine and an adenine at position 12 in all sequences from the 5’SAG2 gene. While it is not possible to discriminate between genotype I and II in the RFLP protocol based on the 5’ end of the SAG2 gene alone (Fazaeli and Ebrahimzadeh 2007), the presence of a double peak in the electropherogram could suggest the presence of a mixed infection with either genotype I or II and genotype III. This has previously been reported in red foxes for several markers used to genotype *T. gondii* (Calero-Bernal et al. 2015). Despite this polymorphism, none of the remaining sequences shared any of the polymorphisms of genotype III, making a mixed infection with this genotype unlikely. Therefore, because all other genes tested were consistent with genotype II, and the SAG2 gene should not be used on its own for the genotyping of *T. gondii* (Fazaeli and Ebrahimzadeh 2007), the sequences of the 5’SAG2 gene were attributed to genotype I/II. Nevertheless, it would be interesting to overcome the limitation of this study by increasing the sampling size, which was constrained by the short sampling timeframe, and by expanding the geographical scope of sampling. This would allow us to see whether other *T. gondii* genotypes are present and to determine if the detected polymorphisms are shared across a broader range of isolates. Analysing the full panel of available genetic markers could also be beneficial in better delineating the genetic diversity of the parasite and, possibly, reveal the presence of atypical strains. Broadening the sampling could also provide further insights into the epidemiology of the parasite, not just in the urban environment, but also in more rural or natural environments where the spread of *T. gondii* may have severe impacts on native marsupial fauna (Dubey 2021).

## Conclusions

This study provides the first insights into the epidemiology and genetic diversity of *T. gondii* in red foxes from Victoria, Australia. Serological screening using the MAT revealed a seroprevalence of 38.9%, while molecular testing confirmed the presence of Type II and Type II-like genotypes. The adaptability of red foxes to diverse environments and their opportunistic foraging behaviour make them useful sentinels for monitoring the distribution of *T. gondii* in Australia. Expanding surveillance to larger sample sizes and wider geographic regions, including varied habitat types, will help to understand differences in the prevalence and transmission dynamics, while comprehensive multilocus genotyping of isolates will further elucidate the diversity and distribution of circulating *T. gondii* strains across the continent.

## Supplementary Information

Below is the link to the electronic supplementary material.

Supplementary Material 1

## Data Availability

All Data is provided within the manuscript and in the supplementary files. Sequencing data produced in this work have been deposited to GenBank under the accession numbers PX493417-PX493423.

## Appendix A. Supporting information

Supplementary data associated with this article can be found in the online version at 10.1007/s00436-025-08606-w.
