# Supplementary material for: Serological and molecular detection of Toxoplasma gondii in naturally infected red foxes (Vulpes vulpes) from Victoria, Australia
Source: Parasitol Res. 2025 Dec 16;124(12):162. doi: 10.1007/s00436-025-08606-w (PMC12711960; doi:10.1007/s00436-025-08606-w)
Supplement: Supplementary file 1 — Supplementary Material 1 [file 436_2025_8606_MOESM1_ESM.docx]

**Supplementary Table 1** Sequences of the primers and probes used in the qPCR assay for the detection of *T. gondii* in tissue samples from red foxes (*Vulpes vulpes*) from Victoria, Australia.

| Marker | Primer name | 5’-3’ Primer sequence |
| --- | --- | --- |
| 529 bp  *T. gondii* | ToxRepF | AGAGACACCGGAATGCGATCT |
|  | ToxRepR | CCCTCTTCTCCACTCTTCAATTCT |
|  | ToxRepProbe | 6FAM-ACGCTTTCCTCGTGGTGATGGCG-IBFQ |
| EHV | EHVFWD | GAT GACACTAGCGACTTCGA |
|  | EHVREV | CAGGGCAGAAACCATAGACA |
|  | EHVProbe | HEX-ACGCTTTCCTCGTGGTGATGGCG-IBFQ |

**Supplementary Table 2** Sequences of the primers and expected nested product size for each of the genes used in the PCR-RFLP protocol to genotype *T. gondii* in tissue samples from red foxes (*Vulpes vulpes*) from Victoria, Australia.

| Gene | Primer name | 5’-3’ Primer sequence | nPCR product size (bp) |
| --- | --- | --- | --- |
| 3’SAG2 | 3’SAG2 Ex F | TCTGTTCTCCGAAGTGACTCC | 222 |
|  | 3’SAG2 Ex R | TCAAAGCGTGCATTATCGC |  |
|  | 3’SAG2 In F | ATTCTCATGCCTCCGCTTC |  |
|  | 3’SAG2 In R | AACGTTTCACGAAGGCACAC |  |
| 5’SAG2 | 5’SAG2 Ex F | GCTACCTCGAACAGGAACAC | 242 |
|  | 5’SAG2 Ex R | GCATCAACAGTCTTCGTTGC |  |
|  | 5’SAG2 In F | GAAATGTTTCAGGTTGCTGC |  |
|  | 5’SAG2 In R | GCAAGAGCGAACTTGAACAC |  |
| GRA6 | GRA6 Ex F | ATTTGTGTTTCCGAGCAGGT | 344 |
|  | GRA6 Ex R | GCACCTTCGCTTGTGGTT |  |
|  | GRA6 In F | TTTCCGAGCAGGTGACCT |  |
|  | GRA6 In R | TCGCCGAAGAGTTGACATAG |  |
| c22-8 | c22-8 Ex F | TGATGCATCCATGCGTTTAT | 521 |
|  | c22-8 Ex R | CCTCCACTTCTTCGGTCTCA |  |
|  | c22-8 In F | TCTCTCTACGTGGACGCC |  |
|  | c22-8 In R | AGGTGCTTGGATATTCGC |  |
| L358 | L358 Ex F | TCTCTCGACTTCGCCTCTTC | 418 |
|  | L358 Ex R | GCAATTTCCTCGAAGACAGG |  |
|  | L358 In F | AGGAGGCGTAGCGCAAGT |  |
|  | L358 In R | CCCTCTGGCTGCAGTGCT |  |

**Supplementary Table 3** Results of molecular detection, with relative Ct value, of red foxes (*Vulpes vulpes*) samples from Victoria, Australia serologically positive to *Toxoplasma gondii*.

| FoxID | qPCR result | Ct value |
| --- | --- | --- |
| Vv01 | + | 27.92 |
| Vv02 | - | N/A |
| Vv03 | + | 26.49 |
| Vv09 | - | N/A |
| Vv11 | - | N/A |
| Vv13 | + | 28.25 |
| Vv15 | - | N/A |
| Vv16 | + | 28.38 |
| Vv17 | + | 29.60 |
| Vv19 | + | 28.78 |
| Vv23 | + | 31.60 |
| Vv24 | + | 25.60 |
| Vv25 | + | 24.54 |
| Vv26 | + | 27.80 |
| Vv36 | + | 26.78 |
| Vv37 | + | 31.94 |
| Vv38 | + | 25.55 |
| Vv39 | + | 25.08 |
| Vv42 | - | N/A |
| Vv43 | - | N/A |
| Vv44 | - | N/A |
| Vv47 | + | 32.89 |
| Vv49 | - | N/A |
| Vv53 | - | N/A |
| Vv54 | + | 29.89 |
| Vv55 | + | 32.36 |
| Vv65 | + | 27.61 |
| Vv69 | - | N/A |
| Vv73 | - | N/A |
| Vv76 | - | N/A |

**Supplementary Material 1:** Justification of the links of the variables in the directed acyclic graph (DAG):

*Sex – Body mass*

Upon reaching adulthood male foxes are generally larger than females in both size (head-body length: 59-90cm in males versus 45-68 cm in females) and body mass (4-14 kg in males versus 3-7 kg in females) (Wilson and Mittermeier 2009).

*Sex – T. gondii seropositivity by MAT*

Males tend to have bigger home ranges compared to females (Wilson and Mittermeier, 2009), are more mobile in their home range compared to females (Servin et al., 1991), and the fact that they reach bigger size and body mass implies they need more food to grow and develop compared to females. These factors, both individually and combined, result in a higher probability of exposure to the parasite in males compared to females.

*Location – Body mass*

Location determines the food availability and therefore the home range of foxes; the more abundant the resources are in the environment, the smaller the home range of a single animal (Wilson and Mittermeier, 2009). Foxes are very adaptable feeders and can benefit from food sources offered either voluntarily or involuntarily by humans in urban settings. Body mass is therefore influenced by location through varying availability of food resources; locations with high productivity will sustain heavier foxes compared to locations where food resources are scarce.

*Location - T. gondii seropositivity by MAT*
Location directly affects type and quantity of food intake and therefore the probability of ingesting either oocyst-contaminated water and vegetable matter, or tissue cysts from infected prey. Furthermore, the degree of environmental contamination is also dependent on the type of environment as oocysts remain infective for longer in cool and moist environments (Dubey 2021).

*Age class – Body mass*Body mass is generally positively related to age class with adult animals being heavier than both kits and juveniles (Wilson and Mittermeier, 2009).

Age class - *T. gondii* seropositivity by MAT
Age class is positively related to *T. gondii* seropositivity because the likelihood of coming in contact with the parasite increases with increasing of age.

**Supplementary Figure 1** Green marks represent red foxes (*Vulpes vulpes*) from Victoria that tested negative to *Toxoplasma gondii* using a modified agglutination test (MAT) (*n* = 47), whereas red marks represent MAT-positive foxes (*n* = 30). Squares were used to indicate foxes from regional Victoria whereas triangles were used to indicate foxes from metropolitan Melbourne. **a**, MAT-negative foxes from regional Victoria; **b**, MAT-negative foxes Metropolitan Melbourne, **c**, MAT-positive foxes from Regional Victoria; **d**, MAT-positive foxes from metropolitan Melbourne.
